# The predictors and patterns of the early recurrence of pancreatic ductal adenocarcinoma after pancreatectomy: the influence of pre- and post- operative adjuvant therapy

**DOI:** 10.1186/s12893-019-0644-z

**Published:** 2019-12-03

**Authors:** Hironobu Suto, Keiichi Okano, Minoru Oshima, Yasuhisa Ando, Shigeo Takahashi, Toru Shibata, Hideki Kamada, Hideki Kobara, Tsutomu Masaki, Yasuyuki Suzuki

**Affiliations:** 1 Department of Gastroenterological Surgery, Faculty of Medicine, Kagawa University, 1750-1, Ikenobe, Kita-gun, Miki-cho, Kagawa 761-0793 Japan; 2 Department of Radiation Oncology, Faculty of Medicine, Kagawa University, Miki-cho, Kagawa Japan; 3 Department of Gastroenterology and Neurology, Faculty of Medicine, Kagawa University, Miki-cho, Kagawa Japan

**Keywords:** Early recurrence, Predictor, Pattern, Preoperative chemoradiotherapy, Neoadjuvant therapy, Adjuvant therapy

## Abstract

**Background:**

The perioperative factors predicting or influencing early pancreatic ductal adenocarcinoma recurrence are unclear. This study attempted to identify the predictive factors for early pancreatic ductal adenocarcinoma recurrence post-pancreatectomy and the influence of pre- and post- operative adjuvant therapy.

**Methods:**

One hundred and fifteen patients undergoing curative resection for pancreatic ductal adenocarcinoma between 2000 and 2016 at our institution were retrospectively analyzed. Patients were divided into two groups: those who did (*n* = 34) and did not (*n* = 81) experience a recurrence within 6 months postoperatively.

**Results:**

Multivariate analyses demonstrated postoperative CA19–9 de-normalization, no postoperative adjuvant chemotherapy, and serosal invasion were independent risk factors for early recurrence (*P* < 0.001, *P* = 0.001, and *P* = 0.010, respectively). A subgroup analysis showed patients with (*n* = 51) and without (*n* = 64) preoperative chemoradiotherapy had different predictors. Although postoperative adjuvant chemotherapy was not a significant indicator in patients with preoperative chemoradiotherapy, CA19–9 de-normalization and no postoperative adjuvant chemotherapy were significant indicators in patients without preoperative chemotherapy. Preoperative chemotherapy strongly prevented early local recurrence while postoperative adjuvant chemotherapy prevented early distant recurrence.

**Conclusions:**

CA19–9 de-normalization was an important predictor of early recurrence of pancreatic ductal adenocarcinoma. Although postoperative adjuvant chemotherapy was an important preventive measure against early recurrence, particularly for distant recurrence, preoperative chemoradiotherapy could strongly prevent the early local recurrence of pancreatic ductal adenocarcinoma. These perioperative adjuvant therapies could have a complementary relationship.

## Background

Pancreatic ductal adenocarcinoma (PDAC) has a poor prognosis; only 3% of patients survive at 5 years after diagnosis [1, 2]. Only 20% of patients with PDAC are eligible to undergo radical resection [3]. Although surgical resection provides the only chance for a cure, it is associated with a median overall survival (OS) period of 11 to 23 months, with a 5-year OS rate of about 20% [4, 5].

Wagner M et al. reported that R0 resection is the most important factor determining outcome in patients with PDAC [6], and Ihsan ED et al. reported that R1 was associated with a decreased OS and disease free survival (DFS) in PDAC when compared with R0 [7]. The early recurrence of PDAC postoperatively is a frequently observed, serious problem, even after microscopically curative resection is performed. La Torre et al. reported that 60% of patients experience local or systemic recurrence within the first 12 months after curative resection [4]. Some reports suggested that the preoperative factors that are associated with the survival time after surgery were tumor size [4], preoperative lymph node metastasis [4], the preoperative serum carbohydrate antigen 19–9 (CA19–9) level [3, 4, 8, 9], histological grades [4, 9], duration of symptoms [3], and the preoperative modified Glasgow Prognostic Score [10]. These might be predictors of the early recurrence of PDAC postoperatively.

Neoadjuvant therapy was not actually recommended for patients with resectable (R)-PDAC in the 2016 National Comprehensive Cancer Network (NCCN) guideline [11], but neoadjuvant chemotherapy or chemoradiotherapy (CRT) may reduce the early recurrence of PDAC. Upfront surgery might be a predictor of the early recurrence of PDAC, even for those with R-PDAC.

The aim of this study was to detect factors influencing on early recurrence and its patterns for the patients with PDAC including neoadjuvant CRT and adjuvant chemotherapy (ACT).

## Methods

This study was approved by the institutional review board of Kagawa University. A total of 142 consecutive patients undergoing pancreatectomy for PDAC between January 2000 and May 2016 were retrospectively examined. Informed consent was obtained from all patients according to the institutional protocol of our hospital. All 142 patients had a PDAC that was histologically examined by at least two pathologists. Of the 142 patients, 27 patients were excluded. Ten patients were censored within 6 months, 10 were classified as unresectable category based on the 2016 NCCN guideline [11], 5 had unclear recurrence timing, 1 underwent R2 resection, and 1 had perioperative mortality. The data from the remaining 115 patients were retrospectively analyzed.

The patients were diagnosed with R (*n* = 86) or borderline resectable ([BR], *n* = 29) PDAC according to the 2016 NCCN guidelines [11]. All surgical procedures were divided into the following three types: classic, pylorus-preserving, or subtotal stomach-preserving pancreaticoduodenectomy (PD) in 75 patients (65%); distal pancreatectomy in 36 (31%); and total pancreatectomy in 4 (3%). Systematic lymph node dissection was performed in all operations. R0 resection was achieved in 104 patients (90%) and R1 was achieved in 11 (10%). R0 resection was defined as negative margin based on Union for International Cancer Control (UICC) definition. Preoperative CRT and postoperative ACT were given to 51 (44%) and 85 (74%) patients, respectively. Among the 115 patients, 34 (30%) experienced early recurrence within 6 months postoperatively (group E) and 81 (70%) did not (group NE).

### Outcome measures

The variables included age; sex; body mass index (BMI); tumor location; resectability; serum C-reactive protein, serum albumin, hemoglobin, and serum CA19–9 levels; neutrophil/lymphocyte ratio (NLR); lymphocyte count; modified Glasgow Prognostic Score [10]; the standardized uptake value (SUV) seen on ^18^F-fluorodeoxy glucose positron emission tomography (FDG-PET); and presence or absence of preoperative CRT. The intraoperative data, including the operation time, estimated blood loss, blood transfusion, and portal vein resection; and postoperative data on morbidity according to the Clavien-Dindo classification [12], CA19–9 normalization status, and postoperative ACT induction, were reviewed and included. A receiver operating characteristic (ROC) curve was constructed to estimate the optimal cutoff value for the recurrence of PDAC within 6 months postoperatively, which was determined as the point closest to the upper left-hand corner of the graph. The ROC curves demonstrated that the cutoff points of the preoperative serum CA19–9 level, NLR, lymphocyte count, SUV on FDG-PET, and tumor size were 173, 4.65, 1648, 4.73, and 3.0, respectively; and the areas under the curve (AUC) were 0.637, 0.466, 0.556, 0.593, and 0.639, respectively.

### Preoperative chemoradiotherapy

We introduced short-term neoadjuvant hypofractionated chemoradiotherapy with S1 in patients with R and BR PDAC between January 2009 and May 2016, and already reported the efficacy and safety [13]. Hypofractionated, external-beam radiotherapy (30 Gy in 10 fractions) with concurrent S1 (60 mg/m^2^) was delivered 5 days per week for 2 weeks prior to pancreatectomy. All of the patients with preoperative CRT (*n* = 51) in this study received radiotherapy in a similar way. Meanwhile, all patients before December 2008 underwent upfront surgery.

### Adjuvant therapy and follow-up

ACT was applied postoperatively unless contraindicated by the patients’ conditions. The patients received gemcitabine, referring to the results of the CONKO-001 trial [14] between 2006 and 2012; or S-1, referring to the results of the JASPAC01 trial since 2013 [15], according to the recommended protocols. S-1 is an oral fluoropyrimidine consisting of tegafur, a prodrug of fluorouracil, and two biochemical modulators. It is characterized by the inhibition of dihydropyrimidine dehydrogenase activity by gimeracil, the maintenance of a high concentration of fluorouracil, and by the suppression of fluorouracil’s phosphorylation in the gastrointestinal tract by oteracil potassium, thereby reducing gastrointestinal toxicity. Gemcitabine at a dose of 1000 mg/m^2^ was administered weekly for 3 weeks, followed by 1 week of rest; oral S-1 (80 mg/m^2^/day) was administered from days 1 to 28, followed by a 2-week rest period or from days 1 to 14, followed by a 1-week rest period. Chemotherapy was initiated within 2 months postoperatively in all patients who were considered eligible for the treatment. The follow-up examinations were performed every 2–3 months for 1 year and every 6 months thereafter, until the disease progressed. Enhanced computed tomography was performed every 6 months. We moved the examination date forward or added magnetic resonance imaging or FDG-PET, if necessary.

### Statistical analysis

The clinicopathological features of patients in the groups E and NE were compared. The categorical variables were compared between the groups using the chi-square test and Fisher’s exact test. Survival was calculated using the Kaplan–Meier method and was compared between the groups using the log-rank test. A multivariate analysis using the backward elimination method of the Cox proportional hazards model was undertaken, using variables from the univariate analysis that were considered to potentially affect survival (*P* < 0.10). *P*-values < 0.05 were considered statistically significant. All statistical analyses were performed using the SPSS Statistics 25.0 for Windows software program (SPSS, Inc., Chicago, IL, USA).

## Results

The median follow-up period was 18 (range: 2–112) months. In the entire group of patients, the median OS period was 24 months, and the 3- and 5-year survival rates were 45.9 and 28.7%, respectively. The median OS times of the groups E (*n* = 34) and NE (*n* = 81) were 9 and 37 months, respectively (*P* < 0.001).

Table 1 shows the results of the univariate and multivariate analyses of the factors that possibly affected the early recurrence of PDAC among the 115 patients. Significant associations with early recurrence were observed for the serum CA19–9 level ≧173 (*P* = 0.026), NLR ≧4.65 (*P* = 0.020), lymphocyte count ≦1648 (*P* = 0.042), SUV on FDG-PET ≧4.73 (*P* = 0.043), postoperative serum CA19–9 de-normalization defined as no return into the normal range (≥37 U/ml) except in patients who had normal preoperative CA19–9 (*P* < 0.001), no postoperative ACT (P < 0.001), pathological tumor size ≧3.0 cm (*P* = 0.028), para-aortic lymph node metastases (*P* = 0.009), and positive serosal (S) and plexus (PL) invasion factors (*P* = 0.009 and *P* = 0.029, respectively). Preoperative CRT did not reach statistical significance in the univariate analysis (*P* = 0.093).

**Table 1:** Univariate and multivariate analyses of the clinical factors affecting the recurrence of PDAC within 6 months after surgery

		Group E (*n* = 34)^b^	Group NE (*n* = 81)^c^	P-value	OR	95% CI	*P*-value^※^
Preoperative variables							
Age^a^	≧ 68	23 (68%)	46 (57%)	0.278			
Sex	Male	19 (56%)	43 (53%)	0.784			
BMI^a^	≦ 21.5	13 (59%)	27 (44%)	0.233			
Tumor location	head	23 (68%)	54 (67%)	0.919			
Resectability	BR	10 (29%)	19 (23%)	0.502			
CRP^a^	≧ 0.18	17 (61%)	32 (43%)	0.103			
CA19–9^a^	≧ 173	21 (62%)	30 (39%)	0.026			
NLR^a^	≧4.65	8 (40%)	10 (16%)	0.020			
Lymphocyte count^a^	≦1648	25 (96%)	58 (79%)	0.038			
mGPS	1 or 2	7 (21%)	13 (17%)	0.382			
SUV in FDG-PET^a^	≧ 4.73	18 (82%)	37 (58%)	0.043			
Preoperative CRT	No	23 (68%)	41 (51%)	0.093			
Intraoperative variables							
Operation time^a^	≧ 609	11 (44%)	39 (59%)	0.197			
Blood loss^a^	≧ 1767	5 (20%)	20 (30%)	0.326			
Transfusion	Yes	5 (25%)	15 (23%)	0.860			
Portal vein resection	Yes	11 (33%)	26 (35%)	0.893			
Postoperative variables							
Morbidity	≧CD3	5 (22%)	13 (22%)	0.948			
Postoperative CA19–9 normalization	No	17 (81%)	9 (20%)	< 0.001	23.10	4.21–126.86	< 0.001
Postoperative ACT	No	18 (53%)	11 (14%)	< 0.001	10.41	2.73–39.64	0.001
Pathological variables							
Tumor size^a^	≧ 3.0	22 (65%)	32 (42%)	0.028			
Histological grade	G2–4	14 (56%)	36 (50%)	0.564			
PALN metastasis	Positive	4 (16%)	1 (2%)	0.009			
LN metastases	Positive	20 (59%)	40 (49%)	0.355			
CY	Positive	2 (9%)	3 (5%)	0.502			
ly	Positive	28 (82%)	57 (70%)	0.182			
v	Positive	32 (94%)	73 (90%)	0.488			
S	Positive	25 (74%)	38 (47%)	0.009	4.942	1.47–16.62	0.010
RP	Positive	26 (76%)	54 (67%)	0.297			
A	Positive	4 (11%)	4 (5%)	0.203			
PV	Positive	10 (29%)	21 (27%)	0.757			
PL	Positive	21 (62%)	32 (40%)	0.029			
Resection status	R1	6 (18%)	5 (6%)	0.056			

※; logistic regression analysis

^a^The cutoff values of age, BMI, CRP, Alb, Hb, CA19–9, NLR, lymphocyte count, SUV on FDG-PET, operation time, blood loss, and tumor size were set by drawing a receiver operating characteristic curve

^b^Recurrence within 6 months after surgery

^c^Recurrence at more than 6 months after surgery

In the multivariate analysis of factors that were found to affect the early recurrence of PDAC in the univariate analysis, postoperative serum CA19–9 de-normalization (odds ratio [OR], 23.10; 95% confidence interval [CI], 4.21–126.86; *P* < 0.001), no postoperative ACT (OR, 10.41; 95% CI, 2.73–39.64; *P* = 0.001) and positive S factor (OR, 4.94; 95% Cl, 1.47–16.62; *P* = 0.010) were independent risk factors for the early recurrence of PDAC. CA19–9 denormalization was seen in 17 of 21 patients (81%) who had preoperative CA19–9 elevation.

Table 2 shows the subgroup analysis results of the factors affecting the early recurrence of PDAC in the 51 patients with preoperative CRT and in the 64 patients with upfront surgery. The percentages of background factors such as resectability (*P* = 0.720), surgical procedure (*P* = 0.756) and Union for International Cancer Control stage (*P* = 0.808) were not significantly different between the E and NE groups (data not shown). Postoperative serum CA19–9 de-normalization (OR, 83.36; 95% CI, 3.32–2095.03; *P* = 0.007) and a positive S factor (OR, 13.08; 95% Cl, 1.25–137.46; *P* = 0.032) were independent risk factors for the early recurrence of PDAC in the multivariate analysis. Postoperative ACT did not significantly affect the early recurrence of PDAC in the multivariate analysis of this subgroup. On the other hand, the results of the univariate and multivariate analyses of the factors affecting the early recurrence of PDAC in the 64 patients who underwent upfront surgery revealed that postoperative serum CA19–9 de-normalization (OR, 39.26; 95% CI, 3.65–422.10; *P* = 0.002) and no postoperative ACT (OR, 15.53; 95% CI, 2.51–96.04; *P* = 0.003) were independent risk factors for early recurrence in this subgroup.

**Table 2:** Subgroup analysis for the clinical factors affecting early recurrence in patients who received CRT preoperatively (*n* = 51) or upfront surgery (*n* = 64)

		Patients who received CRT preoperatively (*n* = 51)						Patients who received upfront surgery (*n* = 64)					
		Group E (*n* = 11)^b^	Group NE (*n* = 40)^c^	*P*-value	HR	95% CI	*P*-value^※^	Group E (*n* = 23)^b^	Group NE (*n* = 41)^c^	*P*-value	HR	95% CI	*P*-value^※^
BMI^a^	< 21.5	7 (70%)	15 (39%)	0.085									
CRP^a^	≧ 0.19							13 (72%)	18 (46%)	0.066			
CA19–9^a^	≧ 173							14 (61%)	15 (38%)	0.088			
NLR^a^	≧ 4.65	6 (60%)	9 (25%)	0.037				2 (20%)	1 (4%)	0.098			
Lymphocyte count^a^	≦ 1648							15 (94%)	25 (66%)	0.032			
Postoperative CA19–9 normalization	No	6 (86%)	5 (22%)	0.002	83.36	3.32–2095.0	0.007	11 (79%)	4 (18%)	< 0.001	39.26	3.65–422.10	0.002
Postoperative ACT	No	5 (45%)	7 (18%)	0.053				13 (57%)	5 (12%)	< 0.001	15.53	2.51–96.04	0.003
Tumor size^a^	≧ 3.0							16 (70%)	16 (43%)	0.047			
PALN metastasis	Positive	1 (10%)	0 (0%)	0.052									
LN metastasis	Positive	8 (73%)	17 (43%)	0.076									
ly	Positive	9 (82%)	21 (53%)	0.080									
S	Positive	10 (91%)	17 (43%)	0.004	13.08	1.25–137.46	0.032						
A	Positive	2 (18%)	1 (3%)	0.050									
PL	Positive	7 (64%)	13 (33%)	0.061									
Resection status	R1							5 (21%)	2 (5%)	0.038	10.03	0.84–118.82	0.067

※; logistic regression analysis, Variables with P ≦ 0.10 are shown in this table

^a^The cutoff values of BMI, CRP, NLR, lymphocyte count and tumor size were set by drawing a receiver operating characteristic curve

^b^Recurrence within 6 months after surgery

^c^Recurrence at more than 6 months after surgery

## Discussion

Recently, the efficacy of preoperative neoadjuvant chemotherapy or CRT for PDAC has been reported, especially for patients with BR- or locally advanced unresectable-PDAC [16–18]. However, neoadjuvant therapy is not recommended for patients with R-PDAC in the NCCN guideline [11], presumably because of insufficient evidence. Preoperative therapy, however, might have a beneficial effect on patient survival in those with R-PDAC through preventing the early recurrence that is often seen even after curative resection.

**Fig. 1:**
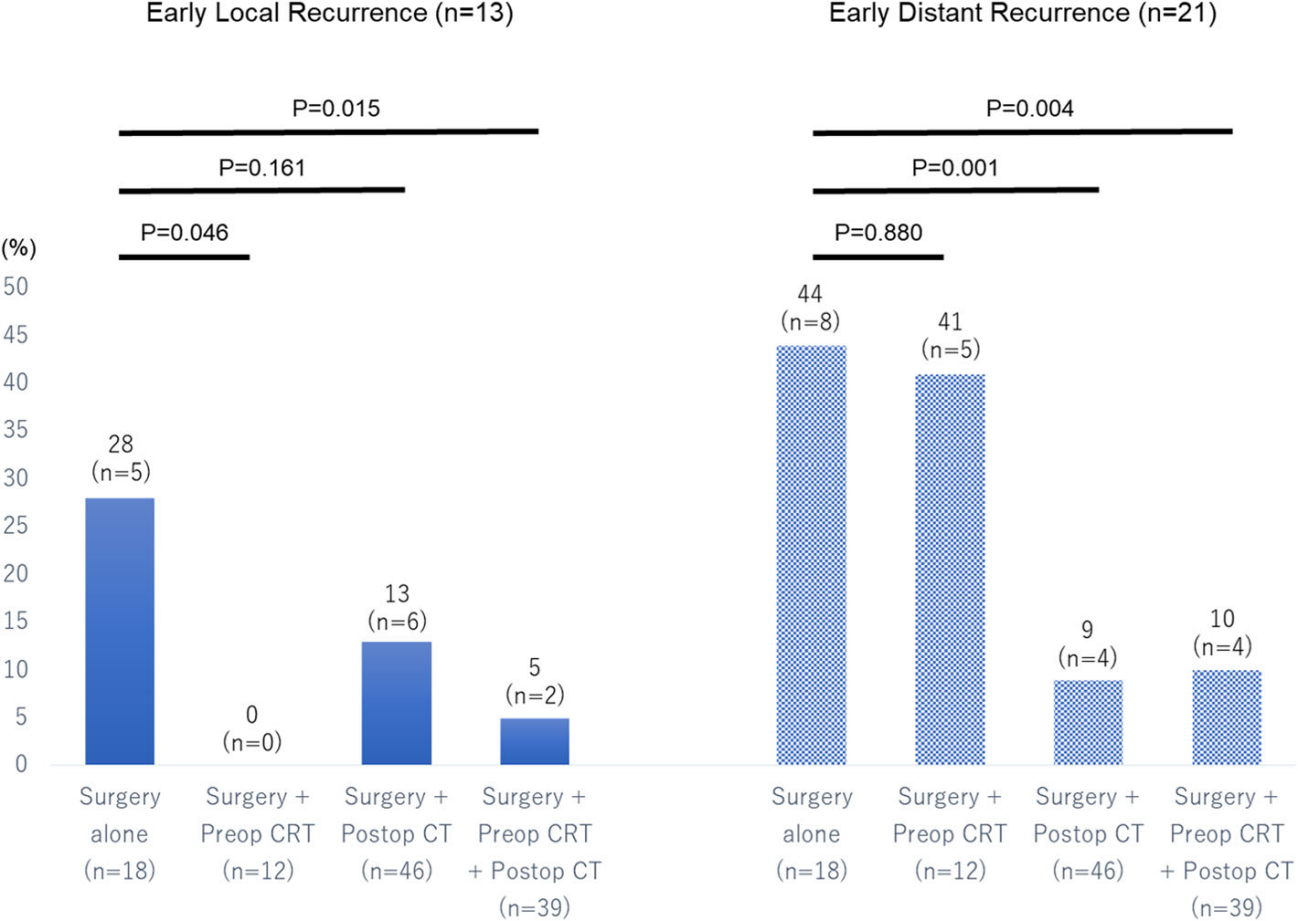
The postoperative early recurrence patterns of PDAC regarding preoperative CRT and postoperative a CRT and postoperative adjuvant chemotherapy. Early local recurrence significantly decreased in patients with preoperative CRT, while early distant recurrence significantly decreased in patients with postoperative chemotherapy

The postoperative recurrence patterns of PDAC in 34 patients in the group E regarding preoperative CRT and postoperative ACT are shown in Fig. 1. The recurrence patterns were defined as the location of the first recurrence. Local recurrence included regional lymph node and plexus nerve recurrence base on radiological findings. We made a final decision while taking account of comprehensive set of factors in terms of tumor marker, FDG-PET and MDCT. In addition, EUS-FNA was applied in several cases with difficult lesion for diagnosis. The recurrence patterns showed that 13 (38%) initially had local recurrence and 21 (62%) patients initially had distant metastasis. Early local recurrence occurred in only 2 patients among a total of 51 patients receiving CRT preoperatively. Even in a group with surgery plus preoperative CRT alone (*n* = 12), no early local recurrence occurred. In contrast, it was seen in 5 (28%) patients undergoing surgery alone (*P* = 0.046) and in 6 (13%) patients with surgery plus postoperative ACT alone. On the other hand, early distant recurrence developed in only 4 (9%) patients with surgery plus postoperative ACT alone. In sharp contrast, it occurred in 8 (44%) who underwent surgery alone (*P* = 0.004) and in 5 (41%) patients with surgery plus preoperative CRT alone. Furthermore, both early local recurrence and distant recurrence significantly decreased in patients who received combined preoperative CRT and postoperative ACT, compared with surgery alone (*P* = 0.015 and *P* = 0.004, respectively). These results clearly demonstrated that preoperative CRT strongly prevented local recurrence but not distant recurrence, and postoperative ACT prevented early distant recurrence but not local recurrence.

The potential advantages of the preoperative delivery of CRT include the ability to sterilize tissues at critical margins oncologically. In the current study, the exposure dose of preoperative CRT was relatively low, at a minimum value of 30 Gy. However, a report from the M.D. Anderson Cancer Center contended that hypofractionated CRT (30 Gy) was associated with margin-negative resection rates, treatment effects, local control, and OS, similar to those associated with standard fractionated CRT (50.4 Gy) [19]. Similar results were proven in our previous study [13] and the current study.

This study demonstrated that the lack of postoperative ACT was a significant predictor of early recurrence. As supported by the results from previous clinical trials [14, 15], ACT is one of the most important factors for preventing early recurrence postoperatively. Judging from the early recurrence pattern that was found in this study, postoperative ACT prevents the early distant recurrence of PDAC, but it might have almost no effect on early local recurrence. Interestingly, there was no significant difference between patients with and without postoperative ACT in those with preoperative CRT. We speculated that preoperative CRT might compensate for a lack of postoperative ACT.

Preoperative CRT itself was not found to be an independent preventive factor against the early recurrence of PDAC in the univariate and multivariate analyses of the entire series of patients. However, the current study demonstrated that preoperative CRT significantly prevented local recurrence. Early local recurrence occurred in only 2 patients among 51 patients receiving CRT preoperatively. Based on these results, preoperative CRT possesses strong efficacy regarding local control and might prevent the early local recurrence of PDAC. In this series, 39 patients were treated with both preoperative CRT and postoperative ACT. In this subgroup, the early recurrence of PDAC occurred in only 6 patients (15%).

The current study has several limitations. This was a retrospective study that was conducted at a single institution. Therefore, the sample size was small and a historical backdrop existed. The types of preoperative examinations such as FDG-PET and postoperative adjuvant therapy changed during the study period. Within the past several years, preoperative CRT has especially been performed for patients with R- and BR-PDAC. Furthermore, there are missing values for several factors in the tables, and the AUCs in the ROC were relatively low and might indicate inadequate cutoff points.

## Conclusions

CA19–9 de-normalization was an important predictor of the early recurrence of PDAC within 6 months after pancreatic resection. Postoperative ACT was an important preventive measure for the early recurrence of PDAC, particularly for distant recurrence. Preoperative CRT had a strong potential to prevent the early local recurrence of PDAC. In addition, preoperative CRT might compensate for the lack of postoperative ACT. In patients who are not expected to be capable of receiving postoperative ACT, preoperative CRT should be considered.

## Data Availability

The datasets used and/or analysed during the present study are available from the corresponding author (HS) on reasonable request.

## Abbreviations

A: Arterial invasion
ACT: Adjuvant Chemotherapy
Alb: Albumin
BMI: Body mass index
BR: Borderline resectable
CA19–9: Carbohydrate antigen 19–9
CD: Clavien-Dindo classification [10]
CI: Confidence Interval
CRP: C-reactive protein
CRT: Chemoradiotherapy
CY: Cytology
FDG-PET: ^18^F-fluorodeoxy Glucose Positron Emission Tomography
Hb: Hemoglobin
HR: Hazard Ratio
LN: Lymph node
ly: Lymph-vessel invasion
mGPS: Modified Glasgow prognostic score [8]
NLR: Neutrophil/Lymphocyte Ratio
OR: Odds Ratio
OS: Overall Survival
PALN: Para-Aortic Lymph Node
PD: Pancreaticoduodenectomy
PDAC: Pancreatic Ductal Adenocarcinoma
PL: Plexus invasion
Postop: Postoperative
Preop: Preoperative
PV: Portal vein invasion
R: Resectable
ROC: Receiver Operating Characteristic
RP: Retroperitoneal invasion
S: Serosal invasion
SUV: Standardized Uptake Value
UICC: Union for International Cancer Control
V: Venous invasion
